# Survival of Severe Acute Respiratory Syndrome Coronavirus 2 (SARS-CoV-2) and Herpes Simplex Virus 1 (HSV-1) on Foods Stored at Refrigerated Temperature

**DOI:** 10.3390/foods10051005

**Published:** 2021-05-04

**Authors:** Janak Dhakal, Mo Jia, Jonathan D. Joyce, Greyson A. Moore, Reza Ovissipour, Andrea S. Bertke

**Affiliations:** 1 Food Science and Technology, Virginia Polytechnic Institute & State University, Blacksburg, VA 24061, USA; jdhakal@vt.edu; 2 Population Health Sciences, Virginia Maryland College of Veterinary Medicine, Center for Emerging Zoonotic and Arthropod-borne Pathogens, Virginia Polytechnic Institute & State University, Blacksburg, VA 24061, USA; moj@vt.edu; 3 Translational Biology Medicine and Health, Center for Emerging Zoonotic and Arthropod-borne Pathogens, Virginia Polytechnic Institute & State University, Blacksburg, VA 24061, USA; jjoyce84@vt.edu; 4 Biomedical and Veterinary Science, Virginia Maryland College of Veterinary Medicine, Center for Emerging Zoonotic and Arthropod-borne Pathogens, Virginia Polytechnic Institute & State University, Blacksburg, VA 24061, USA; victoriam@vt.edu; 5 Food Science and Technology, Agricultural Research and Extension Center, Virginia Polytechnic Institute & State University, Hampton, VA 23669, USA; ovissi@vt.edu

**Keywords:** SARS-CoV-2, COVID-19, HSV-1, foodborne transmission, foodborne illness, food contamination, plaque assay, qPCR, RT-qPCR

## Abstract

Outbreaks of coronavirus infectious disease 2019 (COVID-19) in meat processing plants and media reports of severe acute respiratory syndrome coronavirus 2 (SARS-CoV-2) detection on foods have raised concerns of a public health risk from contaminated foods. We used herpes simplex virus 1, a non-Biosafety Level 3 (non-BSL3) enveloped virus, as a surrogate to develop and validate methods before assessing the survival of infectious SARS-CoV-2 on foods. Several food types, including chicken, seafood, and produce, were held at 4 °C and assessed for infectious virus survival (herpes simplex virus 1 (HSV-1) and SARS-CoV-2) at 0 h, 1 h, and 24 h post-inoculation (hpi) by plaque assay. At all three time points, recovery of SARS-CoV-2 was similar from chicken, salmon, shrimp, and spinach, ranging from 3.4 to 4.3 log PFU/mL. However, initial (0 h) virus recovery from apples and mushrooms was significantly lower than that from poultry and seafood, and infectious virus decreased over time, with recovery from mushrooms becoming undetectable by 24 hpi. Comparing infectious virus titers with viral genome copies confirmed that PCR-based tests only indicate presence of viral nucleic acid, which does not necessarily correlate with the quantity of infectious virus. The survival and high recovery of SARS-CoV-2 on certain foods highlight the importance of safe food handling practices in mitigating any public health concerns related to potentially contaminated foods.

## 1. Introduction

Severe acute respiratory syndrome coronavirus 2 (SARS-CoV-2), the causative agent of the ongoing coronavirus infectious disease 2019 (COVID-19) pandemic, is an enveloped, single-stranded, negative-sense ribonucleic acid (RNA) virus belonging to the family *Coronaviridae*. SARS-CoV-2 is transmitted mainly via respiratory droplets generated during normal activities (e.g., coughing, sneezing, heavy breathing, singing, talking), although airborne transmission has been reported depending on the situational context [1,2,3,4]. In one report, aerosolized feces was suggested to be responsible for several infections in a high-rise apartment building [5]. Transmission via fomites contaminated with virus-laden fluid, especially among household contacts of those infected, has been suggested through case studies but has not been confirmed [6,7]. Given these modes of transmission, the impact of surface contamination with bodily fluids has been a continued area of interest. Previous studies have reported that low-level viable SARS-CoV-2, as determined via tissue culture infectious dose 50% (TCID_50_), can be recovered up to 72 h post-inoculation (hpi) on stainless steel and up to 24 hpi on cardboard held at room temperature with constant humidity under laboratory conditions [7]. These data initially suggested that contamination of common surfaces by those infected with SARS-CoV-2 may present a risk for transmission to susceptible individuals.

Beyond contamination of common surfaces such as packaging materials and counters, SARS-CoV-2 has been recovered at refrigeration (4 °C) and freezing temperatures (0 °C and −80 °C) on some meats and food contact surfaces. These results may be limited in their translation to real world situations as virus recovery was measured with methods less specific than plaque assays (TCID_50_), methods that do not assess for infectious virus (nucleic acid amplification tests), or methods using conditions that are not common to real-world environments (incubation in viral recovery media at constant temperature and humidity) [8,9], the results of which can be misinterpreted. Several press reports regarding the detection of the virus on imported salmon, shrimp, chicken, and ice cream have suggested that food can become contaminated at unknown points along the food supply chain (https://www.foodsafetynews.com/2020/11/china-reports-further-food-related-coronavirus-findings/ accessed on 17 March 2021; https://apnews.com/article/beijing-tianjin-coronavirus-pandemic-wuhan-china-a75ec51c11338190c483c78d65c3348d accessed on 17 March 2021) [10,11]. It is of note that an epidemiological investigation of a cluster of COVID-19 cases in a local market suggested that environmental to human transmission may have occurred between a merchant and SARS-CoV-2-contaminated salmon [12]. However, the risk of contracting SARS-CoV-2 from contaminated foods or food contact surfaces is thought to be minimal (https://www.food.gov.uk/research/research-projects/qualitative-risk-assessment-on-the-risk-of-food-or-food-contact-materials-as-a-transmission-route-for-sars-cov-2 accessed on 17 March 2021; https://www.fda.gov/food/food-safety-during-emergencies/food-safety-and-coronavirus-disease-2019-covid-19 accesed on 17 March 2021; https://www.who.int/publications/i/item/covid-19-and-food-safety-guidance-for-food-businesses accessed on 17 March 2021) [13,14,15] and, according to the United States Department of Agriculture (USDA), the Food and Drug Administration (FDA), and the European Food Safety Authority (EFSA), there is no evidence of food or food packaging being associated with transmission of SARS-CoV-2 (https://www.efsa.europa.eu/en/news/coronavirus-no-evidence-food-source-or-transmission-route accessed on 17 March 2021) [14,16].

However, reported outbreaks of COVID-19 among the staff of major pork and beef processing plants in the United States have raised concerns over the possibility of contracting SARS-CoV-2 from foods and food products contaminated during processing [17,18,19,20]. According to the Food & Environment Reporting Network (FERN), as there is no centralized reporting of COVID-19 cases among food workers available to the public, as of 11 March 2021, at least 1399 meatpacking and food processing plants, as well as 388 farms and production facilities, have reported COVID-19 among a combined 88,203 workers, 376 of whom did not survive their infection (https://thefern.org/2020/04/mapping-covid-19-in-meat-and-food-processing-plants/ accessed on 11 March 2021) [21]. In efforts to maintain the food supply chain, food service employees may be required to continue working in crowded, high stress conditions even when infected (whether symptomatic or presymptomatic), thereby increasing the risk of contamination of food with bodily fluids during food processing and handling [19]. Given that a single cough from an individual shedding SARS-CoV-2 in respiratory fluids can contain >100,000 virus particles and asymptomatic transmission also occurs [22,23,24,25], it is not unrealistic that symptomatic, oligosymptomatic, and asymptomatic individuals could deposit virus on surrounding surfaces, including foods or packaging material. Whether this presents a health risk is unknown. Environmental sampling assessing SARS-CoV-2 surface contamination in 116 food production facilities in non-processing areas revealed the presence of viral RNA in areas with high employee contact, such as tables and breakrooms, and a correlation between surface contamination and positive SARS-CoV-2 test results among food processing personnel was found in one of the food processing plants, indicating that environmental monitoring for SARS-CoV-2 can be used to identify asymptomatic and presymptomatic employees [26]. While this environmental sampling scheme was not designed to assess the presence of infectious virus on surfaces or in foods, it does raise concerns about the risk associated with contaminated food and food contact surfaces [26]. It is worth noting that SARS-CoV-2 is an enveloped virus, and is thus less stable in the environment compared to non-enveloped enteric viruses. Therefore, safe food handling practices and proper cooking should mitigate health risks from contaminated foods. Although the mechanisms of pathogenesis and routes of infection beyond aerosol and droplet transmission are poorly understood, growing evidence suggests that SARS-CoV-2 infects the gastrointestinal tract [27,28]. It is therefore imperative to assess the survival of SARS-CoV-2 on foods.

Disinfection validation, risk assessment modeling, virus transfer through HVAC systems, and conducting research on SARS-CoV-2 require access to biosafety level 3 (BSL-3) facilities, which can be challenging. Thus, identification of an appropriate surrogate virus to use in place of SARS-CoV-2 to investigate the survival of infectious virus on foods would be useful. Similar to SARS-CoV-2, herpes simplex virus 1 (HSV-1) is an enveloped virus that is shed in saliva and nasal secretions and is capable of being expelled in droplets from infected humans. While HSV-1 is not typically considered a foodborne pathogen, previous studies have shown that it can survive on common foods that are routinely touched before consumption [29]. HSV-1 also produces cytopathic effects (CPE) on the same cell type as SARS-CoV-2 (Vero cells), allowing the use of plaque assays to quantify infectious virus on foods in parallel to directly compare HSV-1 and SARS-CoV-2 using the same assay. Therefore, using HSV-1 as a surrogate, we developed methods to recover enveloped viruses from food surfaces. We then assessed the survival of infectious HSV-1 and SARS-CoV-2 on several different categories of food samples kept at refrigerated temperatures (4 °C) for up to 24 h. Thus, we aimed to develop a recovery method for enveloped viruses from foods, assess HSV-1 as a potential surrogate for SARS-CoV-2, and determine the survival of both HSV-1 and SARS-CoV-2 on different types of foods held at refrigeration temperatures.

## 2. Materials and Methods

### 2.1. Cells and Viruses

HSV-1 strain 17+ was originally transferred from John Hay (SUNY Buffalo, NY, USA) to FDA (Bethesda, MD, USA) and propagated in Vero 76 cells (ATCC CRL-1587), and first-passage stocks were transferred to UCSF (San Francisco, CA, USA); first-passage stocks were then transferred to the Bertke lab (Virginia Tech, Blacksburg, VA, USA). Stocks were propagated on Vero 76 cells and titrated by standard plaque assay on Vero 76 cells in quadruplicate to determine concentration. SARS-CoV-2 (Isolate USA-WA1/2020, NR-52281) was procured from BEI, and then propagated and titrated on Vero-E6 cells (ATCC CRL-1586). Both viruses were thawed on ice and diluted in Dulbecco’s modified Eagle medium (DMEM, Fisher Scientific, Waltham, MA, USA) to prepare inocula.

### 2.2. Food Sources and Sample Preparation

Fresh foods were purchased from a local grocery store, maintaining identical brands for each experiment. We selected three broad categories of food types: meat/poultry (chicken), seafood (salmon and shrimp), and produce (spinach, mushroom, and apple). Foods were chopped to approximately 1.5 cm × 1.5 cm and placed into sterile 12 well tissue culture plates or 2.5″ × 5″ × 2.25 mL (1 oz) Whirl-Pak^®^ sampling bags (Nasco, Madison, WI, USA). To mimic contamination of the outer skin of apples, apple skin was used with minimal but uniform flesh. Chicken, salmon, and shrimp were cut into ≈1.7 g pieces. Both the skin/shell and the flesh portions of salmon and shrimp were included in samples, considering this is the standard presentation of these items in the seafood section of most grocery stores.

### 2.3. Virus Inoculation on Foods

Massage Method: Method was modified from [30]. Food samples were placed into Whirl-Pak^®^ bags and inoculated with 10 µL of HSV-1 (1 × 10^6^ PFU). One set of samples was immediately processed (0 h) and two additional sets of samples were incubated at 4 °C for 1 h or 24 h before processing. DMEM (1 mL) was added to each Whirl-Pak^®^ bag, followed by a thorough hand massage to break the food into particles and detach any virus that might have bound to the food or entered into the food matrix. Total fluid from each Whirl-Pak^®^ bag was transferred into microcentrifuge tubes and stored at −80 °C until titration. As a negative control, uninoculated food samples were processed identically.

Rinse Method: Method was modified from [31]. Food samples were placed into sterile 24 well tissue plates and inoculated with 10 µL (2.5 × 10^6^ PFU) or 20 µL (1 × 10^5^ PFU) of HSV-1, or 20 µL (1 × 10^5^ PFU) of SARS-CoV-2 in multiple droplets across the surface of the food samples. One set of samples was immediately processed (0 h) and two additional sets were incubated at 4 °C for 1 h or 24 h. DMEM (1 mL) was used to wash the surfaces of the food samples five times by pipette. Total wash fluid was transferred to microcentrifuge tubes and stored at −80 °C until titration. As a negative control, uninoculated food samples were washed and stored using the same method.

### 2.4. Plaque Assay

To quantify the titer of infectious virus in the wash or massage media, a standard plaque assay was performed on each sample. Samples were serially diluted and inoculated onto confluent Vero E6 or Vero 76 monolayers in 24 well plates in duplicate. The inoculum was incubated for 1 h to adsorb virus and then removed. For HSV-1, Vero 76 cells were used, and inoculum was replaced with DMEM containing 8% fetal bovine serum (FBS), 1% penicillin/streptomycin (PS), and 0.4% pooled human serum to neutralize any virus released into the media. For SARS-CoV-2, Vero E6 cells were used, and inoculum was replaced with a 0.5% agarose overlay to limit the spread of the virus, consisting of DMEM with 8% FBS, 1% P/S, and molecular grade agarose. The inoculum for each experiment was back-titrated by plaque assay, in triplicate, to verify inoculum concentration. The infected plates were incubated at 37 °C with 5% CO_2_ for 48 h, followed by fixation with 10% formaldehyde and staining with plaque dye. Plaques were counted after the plates were dried and the results were expressed as plaque-forming units per mL (PFU/mL) recovered for each sample.

### 2.5. Viral Genome Extraction, Quantitative Polymerase Chain Reaction (qPCR), and Reverse Transcription PCR (RT-PCR)

Viral genome extraction: Viral DNA (HSV-1) or RNA (SARS-CoV-2) was extracted from the virus inoculum and wash medium samples using TRI Reagent LS (ThermoFisher, Waltham, MA, USA) according to the manufacturer’s directions. Briefly, 100 µL of wash medium from each food sample was mixed with an equal volume of TRI Reagent LS Solution. DNA and RNA were separated by the addition of chloroform followed by centrifugation. The top RNA-containing aqueous phase was removed and precipitated in a solution of isopropanol and glycogen for SARS-CoV-2 or discarded for HSV-1. Precipitated RNA was washed post centrifugation with 70% ethanol, air dried, and eluted in molecular-grade water. For HSV-1, DNA in the lower organic layer was precipitated by the addition of 100% ethanol, washed with 0.1 M sodium citrate in 10% ethanol, washed with 70% ethanol, air dried, and eluted in molecular-grade water. Concentrations and purity of the extracted nucleic acids were determined using a NanoDrop 2000 spectrophotometer (ThermoFisher, Waltham, MA, USA) reading absorbances at 260 nm and 280 nm.

HSV-1 qPCR: To determine DNA genome copy number, 10 μL qPCR reactions specific for the thymidine kinase 1 (TK1) gene of HSV-1 using iTaq Universal Probe Supermix (BioRad, Hercules, CA, USA) were run on a ViiA 7 Real-Time PCR system (Applied Biosystems, Foster City, CA, USA), as described previously [32]. The standard setting was used to run the qPCR with the following cycle conditions: 1 cycle of 2 min at 50 °C followed by 10 min at 95 °C; and 40 cycles of 15 s at 95 °C and 60 s at 60 °C. Results were reported as genome copy number per mL of wash media to allow for direct comparison to infectious virus titer measured in PFU/mL.

SARS-CoV-2 reverse transcription polymerase chain reaction (qRT-PCR): To determine RNA genome copy number, 10 μL RT-qPCR reactions specific for the nucleocapsid gene of SARS-CoV-2 (IDT) using the iTaq Universal Probe One-Step Kit (BioRad, Hercules, CA, USA) were run on a ViiA 7 Real-Time PCR system (Applied Biosystems, Foster City, CA, USA), as described previously (https://www.fda.gov/media/134922/download accessed on 11 March 2021) [33]. The standard setting was used to run the qPCR with the following cycle conditions: 1 cycle of 10 min at 50 °C followed by 2 min at 95 °C; and 45 cycles of 3 s at 95 °C and 30 s at 55 °C. Results were reported as genome copy number per mL of wash medium to allow for direct comparison to infectious viral titer measured in PFU/mL.

### 2.6. Statistical Analysis

All experiments were performed three times, in duplicate, with freshly purchased foods each time. Plaque assay data were converted to log PFU/mL prior to statistical analysis. Statistical analyses were conducted using analysis of variance via the GLIMMIX procedure of SAS v. 9.4 (SAS Institute, Cary, NC, USA). Least square means were calculated and significant differences between means were detected at the *p* < 0.05.

## 3. Results

### 3.1. Rinse vs. Massage Method Comparison for Virus Recovery from Foods

Appropriate preparation of food samples for microbiological testing is essential, as the detected microorganisms may be surface contaminants or integrated into the food matrix. Pathogens also possess different properties, allowing them to bind to food surfaces with varying forces [34,35,36]. With minimal information regarding SARS-CoV-2 and its ability to bind to food surfaces or enter the matrices of various foods, we modified and assessed two different methods for optimal recovery of an enveloped virus from foods. We selected a variety of food types, including chicken, apple, and mushroom, and compared a rinse method and a massage or “stomaching” method in parallel. To compare these methods for an enveloped virus, we used herpes simplex virus 1 (HSV-1) at a high inoculum dose as a surrogate virus in place of SARS-CoV-2. Like SARS-CoV-2, HSV-1 is an enveloped virus and is shed in saliva and nasal secretions. HSV-1 has also been found to remain viable on food surfaces following contamination [29].

**Figure 1:**
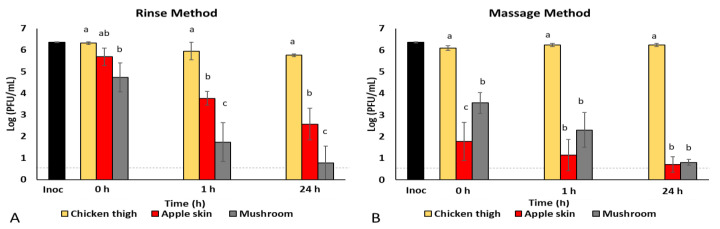
Rinse versus massage method comparison. Herpes simplex virus (HSV-1) was recovered from chicken thigh, apple skin, and mushrooms incubated at 4 °C for 0 h, 1 h, and 24 h using (**A**) rinse method and (**B**) massage method, quantifying infectious virus titer by standard plaque assay on Vero 76 cells and shown as log PFU/mL (*n* = 3). Inoculum (Inoc) titer is shown as PFU/sample (log); 10 µL of a 2.3 × 10^8^ PFU/mL stock (≈2.5 × 10^6^ PFU/sample) were inoculated onto each food sample. The dashed line indicates the detection limit (0.7 log or 5 plaques/mL) of the viral plaque assay. ^a–c^ Means within an incubation time that have a common lowercase letter are not significantly different (*p* < 0.05).

Using the rinse method, in which medium was used to rinse the virus-inoculated food samples, the recovery of HSV-1 from chicken was similar (*p* > 0.05) at 0, 1, and 24 h post-inoculation, ranging from 5.8 to 6.3 log PFU/mL (Figure 1A). The recovery of HSV-1 from apple skin was similar to that of chicken initially (5.7 log PFU/mL at 0 h, *p* > 0.05), but was significantly lower than that of chicken at 1 h (3.8 log PFU/mL) and 24 h (2.6 log PFU/mL) post-inoculation (Figure 1A). In contrast, the initial recovery of HSV-1 from the mushroom was significantly lower than from the chicken (4.7 log PFU/mL at 0 h), as was the case at each of the other time points, with 1.7 log PFU/mL of virus recovered at 1 h and 0.8 log PFU/mL recovered at 24 h post-inoculation (Figure 1A). When the results for each food type were compared over time (Table 1), similar concentrations of HSV-1 were recovered from the chicken (*p* > 0.05) at each time point. In contrast, virus recovered from both the apple skin and mushroom was significantly reduced (*p* < 0.05) at 1 h and 24 h as compared to initial recovery at 0 h (Table 1). Table 1 represents comparative means (±standard deviation) of rinse and massage methods on the recovery of HSV-1 over time in different foods when stored at 4 °C (*n* = 3).

Comparing the rinse vs. massage methods, HSV-1 virus recovery from chicken was similar for both methods, ranging from 5.8 to 6.3 log PFU/mL. However, recovery of virus from apple skin was significantly lower (*p* < 0.05) using the massage method compared to the rinse method. When virus recovery from apple skin was compared, we observed a 3–4 log PFU/mL difference between the methods at 0 and 1 h post-inoculation. Characteristics of apple skin, or potentially factors released from the apple while breaking up the food particles during the massage method, appear to have had a substantial impact on infectious virus recovery. To avoid similar issues with other food types, we selected the rinse method for further evaluation of virus survival on foods.

While using the massage technique, the recovery of HSV-1 from chicken was comparable to that achieved using the rinse technique at each time point of evaluation (Figure 1B). However, the recovery of HSV-1 from apple skin and mushroom was significantly lower (*p* < 0.05) at each time point (0 h, 1 h, and 24 h) compared to that from chicken (Figure 1B). When each food type was compared across time (Table 1), virus recovery from chicken was similar at all time points (*p* > 0.05). Recovery from apple skin was significantly lower than chicken at 0 h post-inoculation (1.8 log PFU/mL) but was not significantly reduced over time (1.1 log PFU/mL at 1 h and 0.7 log PFU/mL at 24 h post-inoculation, Table 1). In contrast, HSV-1 recovery from the mushroom was significantly reduced over time (Table 1).

### 3.2. Survival of HSV-1 on Foods

**Figure 2:**
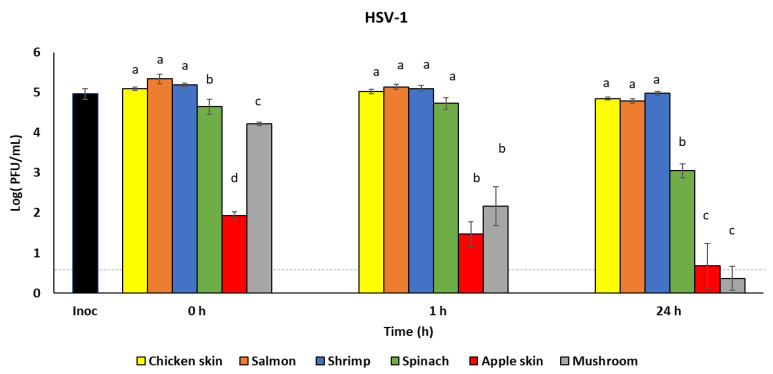
Survival of HSV-1 on foods. HSV-1 was recovered from foods immediately after inoculation (0 h) and at 1 h and 24 h after incubation at 4 °C (*n* = 3). Infectious virus titer was quantified by plaque assay on Vero 76 cells and shown as log PFU/mL. Inoculum (Inoc) is shown as PFU/sample. Dashed line indicates detection limit of the plaque assay (0.7 log pfu/mL). ^a–d^ Means within an incubation time that have a common lowercase letter are not significantly different (*p* < 0.05).

A recent study reported that a patient with a high SARS-CoV-2 load in respiratory fluid (2.35 × 10^9^ copies/mL) may generate 1.23 × 10^5^ copies of viruses from a single cough [25]. To assess infectious virus recovery from food surfaces using a relevant inoculum dose, we inoculated additional food samples with approximately 1 × 10^5^ PFU/mL HSV-1. The virus was recovered using the rinse method immediately after inoculation (0 h) to quantify the maximum virus recoverable from each food type, and after 1 h and 24 h incubation at refrigeration temperature (4 °C). Table 2 shows HSV-1 infectious virus recovery from food samples over time (0 h, 1 h, and 24 h). Reductions in the recovered virus concentrations from chicken skin and salmon were not observed between 0 h and 1 h (*p* > 0.05), while virus concentrations were significantly reduced by 24 h post-inoculation (5.1 and 5.3 log PFU/mL at 0 h to 4.9 and 4.8 log PFU/mL at 24 h (*p* < 0.05), respectively). Similarly, recovery from spinach was significantly reduced by 24 h from 4.7 log PFU/mL at 0 h to 3.1 log PFU/mL at 24 h. Virus concentration recovered from mushroom was significantly reduced from 4.2 log PFU/mL at 0 h to 2.2 log PFU/mL at 1 h (*p* < 0.05), which was further reduced to 0.4 log PFU/mL at 24 h (*p* < 0.05). No reduction in virus concentration from shrimp or apple skin was observed up to 24 h incubation. Nonetheless, initial HSV-1 recovery from apple skin (0 h) was approximately 3 log PFU/mL lower than HSV-1 recovery from chicken skin, salmon, and shrimp. In addition, HSV-1 recovery was compared between the foods at the three different incubation times (Figure 2). At each time point (0 h, 1 h, and 24 h), approximately 5 log PFU/mL of HSV-1 was recovered from each of chicken skin, salmon, and shrimp. HSV-1 recovery from spinach was 0.5–0.7 log PFU/mL lower at 0 h and 1.9–4.7 log PFU/mL lower after 24 h than the HSV-1 recovery from chicken skin, salmon, and shrimp (*p* < 0.05). We also observed that HSV-1 concentrations recovered from mushroom and apple skin were significantly lower than those from chicken skin, salmon, shrimp, and spinach at 0 h, 1 h, and 24 h (*p* < 0.05).

### 3.3. Survival of SARS-CoV-2 on Foods

To validate the use of HSV-1 as a surrogate for SARS-CoV-2 and to assess survival of SARS-CoV-2 on different types of foods, we conducted an identical experiment with SARS-CoV-2, using the rinse method and approximately the same inoculum dose (1 × 10^5^ PFU/mL), methods, and incubation times as we did for HSV-1.

**Figure 3:**
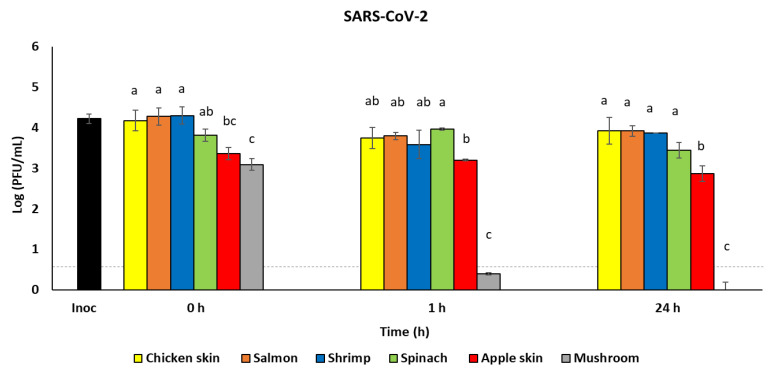
Survival of SARS-CoV-2 on foods. SARS-CoV-2 was recovered from foods immediately after inoculation (0 h) and at 1 h and 24 h after incubation at 4 °C (*n* = 3). Infectious virus titer was quantified by plaque assay on Vero E6 cells and shown as log PFU/mL. Inoculum (Inoc) is shown as PFU/sample. Dashed line indicates detection limit of the plaque assay (0.7 log PFU/mL). ^a–d^ Means within an incubation time that have a common lowercase letter are not significantly different (*p* < 0.05).

At all three time points, the recovery of SARS-CoV-2 from chicken skin, salmon, shrimp, and spinach was similar, ranging from 3.4 to 4.3 log PFU/mL infectious virus (Figure 3, *p* > 0.05). However, initial virus recovery (0 h) from apple skin and mushroom was significantly lower than that from the poultry and seafood (Figure 3, *p* < 0.05). By 1 h post-inoculation, infectious virus recovered from the mushroom was near undetectable (0.8 log PFU/mL), and was significantly lower than all other foods (Figure 3). When the results were compared by specific foods across the different time points, the infectious viral loads recovered from chicken skin, salmon, shrimp, and spinach remained constant over the 24 h (Table 3). The recovery of SARS-CoV-2 infectious virus from apple skin was significantly reduced (*p* < 0.05) at 24 h compared to 0 h. The mushroom showed significantly reduced recovery of SARS-CoV-2 by 1 h post-inoculation; by 24 h post-inoculation, infectious virus was undetectable (Table 3).

### 3.4. Relation of Infectious Virus Titer to Viral Genome Copy Number

The gold standard for confirmation and identification of pathogenic organisms on food is growth of the organism on/in media followed by biochemical testing. Although this is relatively straightforward for foodborne pathogenic bacteria, viruses must be grown in tissue culture and identified using molecular assays. The cell culture and molecular-based methods used for virus identification add time, cost, and complexity to the detection process. Therefore, screening procedures (e.g., swab sampling, rapid antigen tests, or PCR assays) are performed to determine whether viral proteins or viral nucleic acids are present on foods, which do not necessarily equate to the presence of infectious virus. Although many of the recent reports regarding identification of SARS-CoV-2 on foods have not provided sufficient information to determine the methods used to assess the presence of the virus, it is unlikely that food processing plants and food import facilities are assaying for the presence of infectious virus, as they lack the necessary biocontainment facilities, expertise, or time. Therefore, we compared differences between infectious virus titer (as determined by standard plaque assay) with viral genome copy number (as determined by nucleic acid isolation and quantitative PCR or RT-PCR) for both HSV-1 and SARS-CoV-2 from the wash samples obtained from the foods assayed in the previous experiments (Figure 2 and Figure 3).

**Figure 4:**
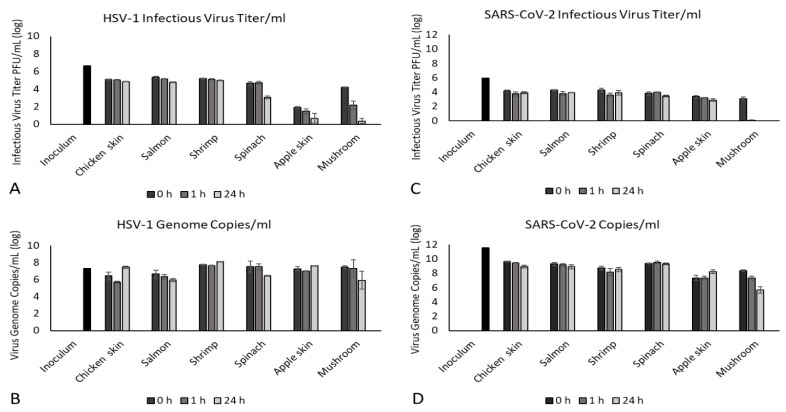
Comparison of infectious virus titer and viral genome copy number. Infectious viral titers were determined by plaque assay for (**A**) HSV-1 on Vero 76 cells, and (**C**) SARS-CoV-2 on Vero E6 cells, shown as log PFU/mL. Viral genome copies were determined by qPCR/RT-PCR for (**B**) HSV-1 by qPCR using primers and probe specific for the HSV-1 thymidine kinase (TK) gene, and (**D**) SARS-CoV-2 by RT-qPCR using primers and probe specific for the SARS-CoV-2 nucleocapsid (N) gene, shown as viral genome copies/mL.

The RNA copy number for SARS-CoV-2 in the inoculum used for the food inoculation studies was 2X greater than the infectious virus titer (11.6 log genome copies/mL vs. 5.9 log PFU/mL, Figure 4C,D). This would suggest that many virions produced during replication of SARS-CoV-2 are not infectious, as would be expected from an RNA virus, given their higher mutation rate despite the unique proofreading capabilities of the SARS-CoV-2 viral RNA dependent RNA polymerase [37]. It is apparent that these noninfectious virions are still detectable by molecular techniques, which has implications for the usefulness of nucleic-acid- and protein-based detection tests that may indicate the presence of viral RNA or protein in the absence of infectious virus. The DNA copy number for HSV-1 in the inoculum used for our contamination studies showed less variation compared to the infectious virus titer (7.3 log genome copies/mL vs. 6.7 log PFU/mL, Figure 4A,B) than did SARS-CoV-2. This is not unexpected given the proofreading capabilities of DNA viruses and their resulting lower mutation rates [38].

In general, the RNA copy number for SARS-CoV-2 followed the overall distribution of infectious virus, with the RNA copy number being ≈2.5× higher than the infectious viral titer (8.2–9.6 log genome copies/mL vs. 3.4–4.3 log PFU/mL, Figure 4C,D) for all foods except apples and mushrooms. Unusually, the RNA copy number of SARS-CoV-2 recovered from apple skin consistently increased over time as the infectious viral titer decreased, which may reflect food-specific effects that are not readily obvious. It is of note that the RNA copy number of SARS-CoV-2 recovered from mushrooms was not related to infectious virus titer. Infectious virus titers declined from 3.1 log PFU/mL at 0 h to undetectable by 24 h; however, viral RNA continued to be detected up to 24 h (0 h: 8.4 log genome copies/mL, 1 h: 7.3 log genome copies/mL, 24 h: 5.7 log genome copies/mL, Figure 4C,D). Moreover, interestingly, not only was infectious virus drastically inactivated over time, but viral RNA was also degraded to lower levels recovered from the mushroom than from any other food tested.

For HSV-1, the DNA copy number generally followed the overall distribution of infectious virus, with the DNA copy number being ≈1.5× higher than the infectious viral titer (5.7–8.1 log genome copies/mL vs. 3.1–5.3 log PFU/mL, Figure 4A,B) for all foods except for apples and mushrooms. The DNA copy number of HSV-1 recovered from apple skin did not increase over time as did the RNA copy number of SARS-CoV-2. It is noteworthy that, as observed for SARS-CoV-2, the DNA copy number of HSV-1 recovered from mushrooms was not related to infectious virus titer. Infectious virus titers recovered from mushrooms declined from 4.2 log PFU/mL at 0 h to 0.4 log PFU/mL by 24 h, although viral DNA continued to be detected up to 24 h (0 h: 7.5 log genome copies/mL, 24 h: 5.9 log genome copies/mL, Figure 4A,B). However, we detected a 75% lower infectious virus titer compared to viral genome copy at 0 h for HSV-1 compared to 54% lower for SARS-CoV-2 at the same time point (HSV-1: 7.3 log copies/mL vs. 1.9 log PFU/mL; SARS-CoV-2: 7.4 log copies/mL vs. 3.4 log PFU/mL, Figure 4). Similarly, we observed a 44% reduction in HSV-1 infectious virus titer at 0 h compared with genome copy number when applied to the mushroom, compared to a 63% reduction in infectious virus titer for SARS-CoV-2 compared to genome copy number at the same time point (HSV-1: 7.5 log copies/mL vs. 4.2 log PFU/mL; SARS-CoV-2:8.4 log copies/mL vs. 3.1 log PFU/mL, Figure 4). This may suggest food-specific effects on the viability of each virus, which may limit the usefulness of viral surrogates for specific food types. These results also confirm that nucleic acid detection methods such as qPCR/RT-PCR do not equate to infectious virus titer, but merely demonstrate presence of viral genetic material.

## 4. Discussion

The presence of gastrointestinal symptoms in COVID-19 patients, including diarrhea, nausea, and vomiting, indicates that SARS-CoV-2 has some level of GI tract involvement [39]. One study found that 48% of COVID-19 patients’ fecal samples evaluated using RT-qPCR were positive for the presence of viral RNA [40]. Direct intragastric inoculation of SARS-CoV-2 in nonhuman primates led to infectious virus isolation from digestive tissues as well as lung, liver, and pancreatic tissues, suggestive of disseminated infection from the GI tract [28]. Similarly to most enveloped viruses, SARS-CoV-2 is inactivated at pH < 3, which is typical of stomach acid (less than pH 3.5) [41]. However, gastric pH can increase to near neutral with a meal, which may permit the virus to survive the stomach if ingested with food [42]. While the mechanism of gut infection is unknown, SARS-CoV-2 has been shown to infect enterocytes as well as intestinal organoids, and to prompt the release of inflammatory cytokines resulting in inflammatory cell recruitment and GI tissue damage [27]. The abundance of angiotensin-converting enzyme 2 (ACE-2), the cellular receptor for SARS-CoV-2, in the oral mucosa, nasal mucosa, nasopharynx, and the gut may provide opportunities for infection through the ingestion of contaminated food, as food would come into direct contact with these tissues during eating [43,44]. Furthermore, a coating of serum proteins on fomites prolongs the infectivity of the virus [45], suggesting that food residues may enhance the survival of SARS-CoV-2 on food, food contact surfaces, or packaging. However, no foodborne cases of COVID-19 have been reported. Although infectious SARS-CoV-2 remains on some foods for at least 24 h, the unlikely chain of events leading to human infection by the consumption of food carrying viable SARS-CoV-2 would require an infected person (symptomatic, asymptomatic, presymptomatic) to expel the virus during the contagious window (2 days prior to onset of symptoms until 7–9 days after) and deposit virus-laden droplets onto the surface of food or packaging during food handling or preparation; another individual could then contract the virus by touching the food or package, transferring the virus to their hands, followed by touching their mouth or nose, or potentially by ingesting contaminated raw or ready to eat food. While it may be possible that consumption of contaminated raw or ready to eat food may lead to viral entry and replication within the mouth or gut, no studies have demonstrated this route of transmission.

The International Organization of Standards (ISO) developed and validated a standard set of methods for extraction of viruses from select foods (ISO 15216-1) (https://www.iso.org/standard/65681.html accessed on 13 April 2021) [46]. This standard utilizes swab sampling to collect virus from food surfaces or an elution-with-agitation approach for soft fruits or leaf and bulb vegetables [46]. However, the methods described in ISO 15216-1 are only validated for the non-enveloped hepatitis A virus (HAV) and norovirus. As an enveloped virus, SARS-CoV-2 may possess different properties for attaching to food surfaces compared to HAV and norovirus. Our goal was to recover the maximum amount of inoculated virus from the foods, rather than to obtain a sampling of the virus on the food surfaces for identification purposes. Therefore, instead of utilizing a swab sampling technique, we compared two commonly used methods for recovering pathogens from foods to determine the optimal method for recovery of an enveloped virus from foods. Rinse methods are commonly used to recover pathogens that are lightly attached to the surface of foods, and have previously been used to recover norovirus from food samples [31]. Massage or “stomaching” techniques are used to detach firmly attached pathogens on or within the matrix of food, as the food is broken into small particles to release the pathogen. This is most commonly used in food challenge studies with pathogenic bacteria such as *Salmonella* or pathogenic *Escherichia coli*, but this method has also been used to recover norovirus from leafy vegetables [30,47]. Since culture methods for HAV and norovirus are nonexistent or not appropriate for application to food matrices, detection under the ISO 15216-1 approach is reliant on RT-PCR detection of the viral genomes, rather than quantification of infectious virus [46]. Therefore, we compared the recovery efficiency of rinse and massage methods using another enveloped virus, HSV-1, as a surrogate. HSV-1 is an enveloped virus and is expelled in oral and nasal secretions, which can be deposited as virus-laden droplets on surfaces. HSV-1 forms CPE on the same cells as SARS-CoV-2, so recovery of infectious HSV-1 and SARS-CoV-2 could be directly compared using the same cell types for plaque assays. This approach aided in the development of methods to recover enveloped viruses from foods and also permitted assessment of HSV-1 as a potential surrogate for SARS-CoV-2.

During our method development and validation processes, we found that the recovery method used (rinse or massage) did not significantly impact the recovery of infectious virus from poultry or seafood up to 24 h post-inoculation, which was not true for fruits and vegetables. This may have been the result of weak attachment of the viruses to the surface of poultry and seafood, allowing full recovery of the virus inoculum. The textures of various foods may also contribute to virus attachment and recovery. Understanding the mechanisms and extent of virus attachment on food surfaces and entry into the food matrices is important for assessing infectious virus persistence and recovery, as well as for implementation of infection control and prevention strategies. The mechanisms by which SARS-CoV-2 attaches to foods have yet to be determined, although these mechanisms have been determined for other foodborne viruses. Norovirus attaches directly to oysters via A-like carbohydrates expressed by the oyster and to produce via electrostatic forces [34,36]. Poliovirus attaches to shellfish through ionic bonding in the shellfish mucus [35]. However, both poliovirus and norovirus are non-enveloped viruses, which likely utilize different mechanisms of attachment than enveloped viruses such as SARS-CoV-2 and HSV-1. Therefore, investigation into attachment mechanisms is needed to better understand how to effectively inactivate or remove enveloped viruses from foods to mitigate risk of infection via food contamination.

In addition, our consistently higher recovery of virus from poultry and seafood compared to fruits and vegetables suggests that these foods provide conditions more favorable to the maintenance of viable viruses for at least 24 h post contamination. For example, protein content in medium, in the form of fetal bovine serum, has been reported to prolong infectivity of SARS-CoV-2 [45]. Thus, high-protein foods may better support viable virus. The neutral pH of these foods (pH 6–7) may also be one such favorable condition contributing to the persistence of infectious virus on poultry and seafood. Furthermore, these foods provide relatively wet surfaces, which may maintain the viability of enveloped viruses like SARS-CoV-2 and HSV-1 by preventing their desiccation and inactivation. While we only assessed the recovery of infectious SARS-CoV-2 from poultry and salmon held at 4 °C up to 24 h, our findings concur with a recent preprint reporting similar findings in poultry, pork, and salmon held at 4 °C, −20 °C, and −80 °C for up to 21 days [48]. Freezing temperatures are known to maintain viability of viruses for extended periods of time (years). Although assessment of how long SARS-CoV-2 survives on foods at freezing temperatures may be of interest, we would argue that consumption of poultry, meats, and seafood held at 4 °C for 21 days would be inadvisable and therefore, detection of infectious virus on foods held at refrigeration temperatures beyond a few days is irrelevant for human risk of infection.

Both viruses were relatively stable on spinach over the 24 h incubation period (HSV-1: 4.7–3.0 log PFU/mL, SARS-CoV-2: 3.8–3.4 log PFU/mL). This is not surprising given that viable human (229E up to 2 days) and nonhuman (bovine coronavirus up to 14 days) coronaviruses have been recovered on lettuce stored at 4 °C [48,49]. Interestingly, bovine coronavirus viral RNA was detected up to 30 days, again demonstrating the disconnection between nucleic-acid-based detection systems and infectious virus assays [49]. Similar studies assessing the stability of HSV-1 on these vegetables have not been published.

We recovered significantly less infectious HSV-1 from apple skin and mushrooms compared to poultry and seafood, using both massage and rinse techniques. The massage technique, which generated an extract of apple juice, reduced the infectious viral titer of HSV-1 to a greater extent than the rinse method, which contained minimal apple juice, indicating that the presence of apple juice may have had an antiviral effect. This reduction in infectious virus may be due to the previously reported antiviral activity of apple juice, as observed against poliovirus type 1, hepatitis A virus, and coxsackievirus B5 [50,51]. While the antiviral activity of apple juice may be due to its acidic pH (3.35–4), Konowalchuk and Speirs showed that apple juice had greater antiviral activity at neutral pH [50], suggesting that other factors in apple juice may play a role. For example, antiviral effects of apple pomace extract against HSV-1 and HSV-2 have been attributed to flavonoid components [52]. It is noteworthy that when the concentration of the viral inoculum was reduced to be more representative of the typical concentration of virus expelled during a cough, a similar amount of infectious HSV-1 was recovered using both methods (comparing Figure 2 to Figure 1B), suggesting that the antiviral activity of apples is also partially dependent on the concentration of the viral inoculum. Apples have also been reported to have a rapid antiviral effect against other coronaviruses, as a previous study demonstrated that no infectious human coronavirus 229E was detected after 24 h when the initial inoculum dose was approximately 10^4^ PFU/mL [53]. However, we did not observe a similar antiviral effect of apples producing a reduction of infectious SARS-CoV-2, demonstrating that reduction in infectious viral titers is variable between different viruses. Furthermore, SARS-CoV-2 appears to be resistant to the intrinsic antiviral activity of apples.

Mushrooms, however, demonstrated robust reductions in infectious viral titers for both HSV-1 and SARS-CoV-2, reducing viable HSV-1 to < 1 log PFU/mL within 24 h and reducing viable SARS-CoV-2 to <1 log PFU/mL within 1 h. During the method development process, a slightly greater initial reduction in infectious HSV-1 was observed when using the massage method vs. the rinse method (3.6 vs. 4.7 log PFU/mL at 0 h), although this was not observed at later time points. This observation may suggest the release of substance(s) from the mushroom that contributed to this initial reduction in infectious virus. Previous reports using cell culture have shown that extracts from various mushroom types have some level of antiviral activity against HIV, influenza A and B, HSV-1 and HSV-2, and hepatitis B and C viruses [54]. This antiviral activity may be due in part to ganodermadiol, a sterol in mushrooms, which has been shown to be effective against HSV-1 [55]. Interestingly, not only did infectious SARS-CoV-2 titers decline drastically at 1 h and become undetectable by 24 h, but viral RNA was also degraded by compound(s) contained in/on mushrooms. This pattern was also reproducible with HSV-1 and merits further study, as compounds in mushrooms appear to be able to destroy both infectious virus and viral nucleic acids fairly rapidly.

When infectious virus titer, determined via standard plaque assay, was compared to viral genome copy number, determined via qPCR and RT-qPCR, a trend was observed for poultry and seafood, as well as for spinach, with viral genome copy numbers being ≈1.5–2.5× greater than infectious viral titer for HSV-1 and SARS-CoV-2, respectively. While this trend of genome copy number and viable virus titer was observed for these food groups, it is uncertain how translatable this trend would be to other food types, other time points, or other storage conditions, and it should therefore not be interpreted as a predictive relationship. This trend was not observed for either virus when mushrooms or fruit were inoculated. The rapid decline in viable SARS-CoV-2 from 3.1 log PFU/mL at 0 h to undetectable by 24 h, with continued detection of >5 log genome copies/mL viral RNA, highlights the limitations of PCR-based testing if the goal of testing is to determine whether a food item is contaminated with infectious virus. It is possible to detect ≈5 log genome copies/mL in complete absence of infectious SARS-CoV-2. The fact that this pattern was also reproducible with HSV-1-inoculated mushrooms (5.9 log genome copies/mL detected, 0.4 log PFU/mL infectious virions detected) lends further support to this observation. These observations suggest that no standard predictive relationship exists between food type and retention of infectious virus that can be broadly applied across food groups/items. Other findings also support the idea that that norovirus, hepatitis A virus, and human coronavirus 229E that are not infectious could still present intact RNA on foods, resulting in positive RT-PCR results [53,56,57]. These results confirm the virological canon that nucleic acid amplification tests (e.g., PCR, qPCR, RT-qPCR) report on the presence of viral nucleic acid, which does not necessarily correlate to the presence of viable infectious virus. Although methods described in the International Organization for Standardization (ISO) 15216-1 are widely used and accepted for virus recovery and quantification from food surfaces, these methods are only validated for detection of the presence of the viral genomes of non-enveloped viruses such as hepatitis A and norovirus [46]. If the goal of environmental monitoring of food samples and food contact surfaces is to determine whether a food or surface has become contaminated with infectious virus that may pose a public health risk, qPCR and RT-qPCR may be used as an initial screen, but a follow-up test for infectivity is needed. Although positive nucleic acid tests would likely prompt disinfection and decontamination of food processing facilities, these procedures are costly, cause delays in production, and may result in food waste through disposal of food thought to be contaminated, but which may not actually carry infectious virus. As the vast majority of farms, food processing plants, and import facilities do not have access to the biocontainment facilities, expertise, or time needed to perform cell culture assays to confirm the presence or quantity of infectious virus, this represents a much-needed area of research to provide the food industry with rapid, inexpensive, and reliable assays to confirm infectious virus in food samples, not just the presence of nucleic acids that may not pose a health risk.

We acknowledge several limitations of our studies. First, we tested survival of SARS-CoV-2 on only six different foods. Our focus was on survival of the virus on a broad range of food types to gain an initial understanding of the types of foods that may pose a public health risk. Future studies will more comprehensively address common foods, particularly those consumed raw or purchased ready to eat. Second, we only tested survival through 24 h. Considering high titers of infectious virus remained on the chicken and seafood at 24 h, understanding precisely how long the virus remains viable on foods is necessary. Although the foods with the highest recovery, chicken and seafood, would likely be cooked and consumed within a few days of storage at 4 °C, a variety of foods are held at refrigeration temperatures for longer periods of time. Therefore, additional studies of SARS-CoV-2 viability on relevant foods held at refrigeration temperatures for longer periods of time are needed. Finally, it is important to note that interpretation of these results requires caution. While these results answer our original research question of whether viable HSV-1 and SARS-CoV-2 can be recovered from some types of foods held in cold-chain conditions, it does not answer questions about the infection potential of virus-contaminated foods to humans. As an enveloped virus, which is less stable in the environment than non-enveloped enteric viruses, cooking temperatures inactivate SARS-CoV-2, so properly cooked foods should be safe for eating [58]. These results should not be interpreted as indicating the amount of viable virus recovered from these foods that is directly infectious to humans, is capable of replication in the human alimentary tract, can produce gastrointestinal disease, or can produce systemic disease. These are pertinent research questions which require further study to address.

## 5. Conclusions

Overall, viable HSV-1 and SARS-CoV-2 were recovered at comparable levels from poultry and seafood held in cold-chain conditions for up to 24 h. The relative stability of each virus on these food types may have been in part due to the moisture or protein contents of the foods. Recovery of viable HSV-1 and SARS-CoV-2 diverged when applied to produce, however, with apples displaying a significant antiviral effect for HSV-1 but not for SARS-CoV-2. Mushrooms exerted a profound antiviral effect, reducing HSV-1 infectious virus to less than 10 infectious virus particles over 24 h and ablating SARS-CoV-2 to undetectable levels within 1 h. Thus, intrinsic characteristics or components of some types of produce demonstrate antiviral activity, which varies by food type and virus. Our studies suggest that HSV-1 can serve as a reasonable surrogate for assessing survival of SARS-CoV-2 on some foods, but not others. Furthermore, we confirm that PCR-based assays for presence of virus on foods may be used as a screening tool but confirmatory studies must be performed to assess viable infectious virus, as no predictive relationship exists between genome copy number and infectious virus particles on various types of food. Our findings highlight the importance of safe food handling and storage practices, environmental monitoring, and the consistent use of personal protective equipment in poultry and seafood processing facilities as well as in grocery stores, in order to prevent accidental contamination of foods. Although it is not clear which factors within various foods dictate how long infectious virus remains viable, future studies will be expanded to include additional food types and broader storage conditions to determine what food types under what storage conditions allow persistence of SARS-CoV-2 on foods.

## Figures and Tables

**Table 1:** HSV-1 Rinse vs. Massage.

Virus Concentrations (log PFU/mL) over Time (Hours Post-Inoculation)						
	Rinse			Massage		
Inoculum	8.4 ± 0.4 *	-	-	-	-	-
	0 h	1 h	24 h	0 h	1 h	24 h
Chicken thigh	6.3 ± 0.1 ^a^	6.0 ± 0.7 ^a^	5.8 ± 0.1 ^a^	6.1 ± 0.2 ^A^	6.2 ± 0.1 ^A^	6.2 ± 0.1 ^A^
Apple skin	5.7 ± 0.7 ^a^	3.8 ± 0.5 ^b^	2.6 ± 1.3 ^b^	1.8 ± 1.5 ^A^	1.1 ± 1.2 ^A^	0.7 ± 0.6 ^A^
Mushroom	4.7 ± 1.2 ^a^	1.7 ± 1.6 ^b^	0.8 ± 1.3 ^b^	3.6 ± 0.8 ^A^	2.3 ± 1.4 ^AB^	0.8 ± 0.2 ^B^

Least square means ± standard deviation of HSV-1 concentrations from inoculum or foods (*n* = 3). * Concentration of inoculum/mL. 10 µL (2.5 × 10^6^ PFU) was applied to each food sample. Lower-case superscripts (a,b) statistically compare the rinse method, and upper-case superscripts (A,B) compare the massage method across the time points. Foods that have common case letters were not significantly different (*p* < 0.05) in terms of virus recovery.

**Table 2:** HSV-1 Recovery from Foods.

Virus Concentrations (log PFU/mL) over Time (Hours Post-Inoculation)			
Inoculum	6.7 ± 0.2 *	-	-
	0 h	1 h	24 h
Chicken skin	5.1 ± 0.1 ^a^	5.0 ± 0.1 ^ab^	4.9 ± 0.1 ^b^
Salmon	5.3 ± 0.2 ^a^	5.1 ± 0.1 ^ab^	4.8 ± 0.1 ^b^
Shrimp	5.2 ± 0.1 ^a^	5.1 ± 0.1 ^a^	5.0 ± 0.1 ^a^
Spinach	4.7 ± 0.3 ^a^	4.7 ± 0.3 ^a^	3.1 ± 0.3 ^b^
Apple skin	1.9 ± 0.2 ^a^	1.5 ± 0.5 ^a^	0.7 ± 1.0 ^a^
Mushroom	4.2 ± 0.1 ^a^	2.2 ± 0.8 ^b^	0.4 ± 0.5 ^c^

Least square means ± standard deviation of HSV-1 concentrations from inoculum or foods (*n* = 3). * Concentration of inoculum/mL. 20 uL (≈1 × 10^5^ PFU) was applied to each food sample. ^a–c^ Lower-case superscripts statistically compare each food across the time points. Foods that have a common lowercase letter are not significantly different (*p* < 0.05) in terms of virus recovery.

**Table 3:** SARS-CoV-2 Recovery from Foods.

Virus Concentrations (log PFU/mL) over Time (Hours Post-Inoculation)			
Inoculum	5.9 ± 0.2 *	-	-
	0 h	1 h	24 h
Chicken skin	4.2 ± 0.2 ^a^	3.7 ± 0.5 ^a^	3.9 ± 0.3 ^a^
Salmon	4.3 ± 0.1 ^a^	3.8 ± 0.5 ^a^	3.9 ± 0.1 ^a^
Shrimp	4.3 ± 0.4 ^a^	3.6 ± 0.4 ^a^	3.9 ± 0.6 ^a^
Spinach	3.8 ± 0.4 ^a^	4.0 ± 0.1 ^a^	3.4 ± 0.2 ^a^
Apple skin	3.4 ± 0.3 ^a^	3.2 ± 0.0 ^ab^	2.9 ± 0.3 ^b^
Mushroom	3.1 ± 0.4 ^a^	0.1 ± 0.1 ^b^	0.0 ± 0.0 ^b^

Least square means ± standard deviation of HSV-1 concentrations from inoculum or foods (*n* = 3). * Concentration of inoculum/mL. 20 uL (≈1 × 10^5^ PFU) was applied to each food sample. a,b Lower-case superscripts statistically compare each food across the time points. Foods that have a common lowercase letter were not significantly different (*p* < 0.05) in terms of virus recovery.

## Data Availability

Data is available upon request.
